# Perceptions and clinical use of biosimilars among rheumatologists in ArLAR countries: a cross-sectional survey

**DOI:** 10.3389/fmed.2026.1780691

**Published:** 2026-03-23

**Authors:** Asal Adnan, Nabaa Ihsan Awadh, Yasameen Abbas Humadi, Faiq I. Gorial, Nelly Ziade, Ali A. Younis, Ihsane Hmamouchi, Lina El Kibbi, Hussein Halabi, Chafika Haouichat, Basel K. Masri, Enas Osama Hassan Omer, Aya Ahmed Shimal, Ahmed Dheyaa Al-Obaidi, Nizar Abdullateef Jassim

**Affiliations:** 1 Rheumatology Unit, Department of Medicine, University of Baghdad, Baghdad, Iraq; 2 Department of Internal Medicine, Al-Nahrain University, College of Medicine, Baghdad, Iraq; 3 Department of Rheumatology, Hotel-Dieu Hospital, Saint Joseph University, Beirut, Lebanon; 4 Department of Medicine, College of Medicine, University of Mosul, Mosul, Iraq; 5 Health Sciences Research Center, Faculty of Medicine, International University of Rabat, Rabat, Morocco; 6 Division of Rheumatology, Department of Internal Medicine, SMC Healthcare, Riyadh, Saudi Arabia; 7 Department of Medicine, Section of Rheumatology, King Faisal Specialist Hospital and Research Center, Jeddah, Saudi Arabia; 8 Faculty of Medicine El Mahdi Si Ahmed, University Saad Dahlab-Blida 1, Blida, Algeria; 9 Jordan Hospital, Amman, Jordan; 10 College of Medicine, University of Bahri, Khartoum, Sudan; 11 Iraqi Board for Medical Specializations, Baghdad, Iraq

**Keywords:** ArLAR, biologic therapy, biosimilars, cross-sectional survey, healthcare policy, physician perceptions, regulatory approval, rheumatology

## Abstract

**Background:**

Biosimilars are increasingly used in rheumatology practice; however, knowledge gaps and regulatory uncertainties may affect their acceptance. This study assessed the knowledge, perceptions, and clinical practices related to biosimilars among rheumatologists in the Arab League of Associations for Rheumatology (ArLAR) countries.

**Methods:**

A web-based survey of 19 questions, adapted from a European Society for Medical Oncology (ESMO) tool, was distributed among ArLAR-affiliated rheumatologists between July 2024 and January 2025. The questionnaire assessed knowledge, attitudes, clinical use, and educational preferences. A French version was developed and validated by the study steering committee. Responses were analyzed using descriptive statistics.

**Results:**

A total of 105 rheumatologists from various Arab countries participated in the survey. The mean age of participants was 43.6 ± 11.7 years, and 61.9% were females. Most participants were adult rheumatologists (94.3%), primarily from Iraq (37.1%), Morocco (19%), and Algeria (16.2%). While 85.7% reported using biosimilars in practice, only 50.5% correctly identified the formal definition. Comfort with EMA/FDA-approved biosimilars was high (mean score 4.22/5), while concerns remained about switching in stable patients and among multiple biosimilars. The majority (80%) expressed interest in additional biosimilar education, particularly on regulatory approval, extrapolation, and safety.

**Conclusion:**

ArLAR rheumatologists show high uptake of biosimilars but moderate understanding of key regulatory and clinical concepts. Regionally adapted educational programs are needed to support evidence-based biosimilar use and improve confidence in clinical decision-making.

## Highlights

- This is the first ArLAR-wide survey assessing rheumatologists’ perceptions and clinical use of biosimilars.
- The findings show variability in biosimilar familiarity, comfort, and prescribing practices across countries.
- Economic concerns and regulatory environments significantly influence biosimilar uptake in the region.
- The study identifies educational and policy gaps and proposes region-specific strategies for improving biosimilar adoption.

## 1 Introduction

Globally, rheumatology practice now faces both major opportunities and challenges due to the growing availability of biosimilar medications. Biosimilars, which are defined as biologic products that are highly similar to an already-approved reference biologic in terms of quality, efficacy, and safety, provide a potential remedy for the growing expense of biologic therapies, especially in low-income and lower-middle-income nations, thereby enhancing patient access to necessary treatments (1). In rheumatology, where chronic inflammatory diseases often require long-term biologic intervention, the strategic integration of biosimilars into clinical practice has been emphasized by multiple international recommendations, including those from the European Alliance of Associations for Rheumatology (EULAR) (2) and the American College of Rheumatology (ACR) (3). Despite favorable regulatory frameworks and mounting empirical data showing that biosimilars’ efficacy, safety, and immunogenicity profiles are similar to those of their reference products (4) physician adoption remains variable. Surveys among rheumatologists and other medical specialists in Europe, North America, and Asia have consistently highlighted concerns regarding interchangeability, extrapolation of indications, immunogenicity, and patient acceptance as significant barriers to biosimilar use (5–8). Significantly, it has been demonstrated that patient acceptance and treatment outcomes are influenced by the prescriber’s confidence and communication regarding biosimilars. According to new research, the nocebo effect—a phenomenon where unfavorable treatment expectations result in worse outcomes—may be lessened by doctor support and assurance, which would increase biosimilar therapy adherence and perceived efficacy (9, 10). This underscores how physician expertise and communication style play dual roles in shaping patient responses and biosimilar uptake.

Adoption of biosimilars is increasing in the Arab world, but it remains uneven across nations due to differences in procurement practices, regulatory frameworks, and levels of clinical education. Many Arab-based pharmaceutical companies, including Julphar (UAE), Hikma (Jordan), and Algerian Biocare, have entered the biosimilars market, contributing to local production capacity and possibly improving affordability and supply sustainability. Biosimilars are available for multiple indications in the majority of Arab League countries (11). However, published data on biosimilar penetration in rheumatology across the region remain scarce. A better understanding of physician practices and preferences is therefore crucial for supporting wider implementation.

To date, no comprehensive study has examined the perceptions, clinical experiences, and knowledge levels of rheumatologists practicing in ArLAR-affiliated countries regarding biosimilar use. The Arab League of Associations for Rheumatology (ArLAR) is a regional scientific organization that brings together national rheumatology societies across the Arab world, including countries from the Gulf Cooperation Council (GCC), the Levant, and North Africa. ArLAR aims to promote rheumatology education, research collaboration, and harmonization of clinical practice across member states. Given the unique regulatory landscapes, procurement strategies, and patient care dynamics within these countries, region-specific data are essential for informing targeted educational initiatives and harmonizing biosimilar integration strategies. This study was thus designed to evaluate the knowledge, clinical experience, and perceptions of rheumatologists across ArLAR countries regarding biosimilar medicines. Additionally, it aimed to identify key concerns, perceived barriers, and priorities for future educational efforts aimed at promoting biosimilar adoption in rheumatology practice.

## 2 Materials and methods

### 2.1 Study design and participants

This was a descriptive, cross-sectional study based on a web-based survey conducted among rheumatologists practicing in ArLAR-affiliated countries. Participants included adult and pediatric rheumatologists, as well as internists involved in rheumatologic care.

### 2.2 Survey instrument

The survey comprised 19 items derived from a previously validated questionnaire developed by the European Society for Medical Oncology (ESMO), which was adapted for use in rheumatology practice (8). Further modifications were undertaken to address regional context and terminology. The finalized version of the questionnaire was evaluated and endorsed by an expert committee appointed by ArLAR. To facilitate participation from francophone countries in North Africa, a French version was produced through translation by bilingual committee members. Careful harmonization was performed to maintain conceptual equivalence and consistency in response options across the Arabic/English and French versions. The questionnaire assessed several domains related to biosimilar use, including foundational knowledge, perceptions of efficacy, safety and immunogenicity, attitudes toward extrapolation, interchangeability and switching, current prescribing practices, as well as educational needs and perceived barriers to prescription. Question formats included multiple-choice items (including knowledge-based questions), multiple-response checkbox items, Likert-scale questions (rated on 1–5 or 1–10 scales) evaluating agreement, confidence or perceived importance, and an optional open-ended item for additional comments or suggestions. The complete survey instrument is provided as Supplementary material S1.

### 2.3 Survey distribution, sampling method and data integrity

The survey was distributed through the Google Forms platform and disseminated via ArLAR’s social media networks. The data collection period began in July 2024 and ended in January 2025. A reminder was issued at the midpoint of the data collection. Participants were recruited using a convenience sampling method. Due to the online and anonymous nature of the survey, an exact response rate could not be calculated, as the total number of individuals who received or viewed the invitation is unknown. To minimize duplicate submissions, the Google Forms platform was configured to restrict responses to one per account. Additionally, responses were screened for consistency and completeness. Data were exported to Microsoft Excel and analyzed using IBM SPSS Statistics version 26.0. Participation was voluntary and anonymous.

### 2.4 Ethical considerations

This study involved human participants and was conducted in accordance with the ethical principles of the Declaration of Helsinki. Ethical approval was obtained from the Ethics Committee of Internal Medicine, Baghdad Teaching Hospital. Where required, additional approvals were obtained from local Institutional Review Boards in participating ArLAR countries in accordance with national regulations. All participants provided informed electronic consent prior to participation. Participation was voluntary and anonymous.

### 2.5 Statistical analysis

Survey responses were analyzed using descriptive statistical methods. Biosimilar knowledge among participating rheumatologists was assessed using a predefined scoring approach based on established educational validation principles. The knowledge score was calculated based on four objective multiple-choice items: (1) correct identification of the regulatory definition of a biosimilar, (2) understanding of the concept of extrapolation of indications, (3) identification of the most appropriate endpoint for comparative biosimilar trials, and (4) knowledge of the biosimilar development and approval process. Each correct response was assigned one point, resulting in a total possible score ranging from 0 to 4. A threshold of at least 75% correct responses (corresponding to 3 out of 4 knowledge-based items) was considered indicative of adequate knowledge, in accordance with the competency-based evaluation framework described by De Ketele and Gérard (12). This threshold was selected because achieving at least three-quarters of correct responses is widely considered indicative of satisfactory mastery in competency-based clinical education frameworks, reflecting sufficient conceptual understanding for safe clinical application. For example, a respondent correctly answering three of the four items received a score of 3 (75%) and was categorized as having “adequate knowledge,” whereas a respondent with two correct responses (50%) was categorized as having “limited knowledge.”

## 3 Results

### 3.1 Participant characteristics

This survey included rheumatologists from 13 different ArLAR countries, with a total of 105 participants. The mean age was 43.6 ± 11.7 years (range: 29–74), and 61.9% were females. The majority were adult rheumatologists (94.3%), with a small representation of pediatric rheumatologists (4.8%) and internists (0.9%) (Table 1). Most respondents practiced in Iraq (37.1%), followed by Morocco (19%) and Algeria (16.2%). Other participating countries included Egypt, Palestine, Tunisia, Jordan, Syria, Lebanon, Kuwait, UAE, and Oman. Over half of the participants (54.3%) had 1–10 years of practice experience, while 21.0% had 11–20 years, and 24.7% had been practicing for more than 20 years. The mean duration of clinical practice was 14.2 ± 10.9 years.

**Table 1:** Baseline characteristics of the participated physicians (*n* = 105).

Variable	Frequency	Percent
Age		
≤35	41	39.0
36–50	36	34.3
>50	28	26.7
Mean ± SD	43.6 ± 11.7	
Range	29–74	
Gender		
Male	40	38.1
Female	65	61.9
Speciality		
Adult rheumatologist	99	94.3
Pediatric rheumatologist	5	4.8
Internist	1	0.9
Country of practice		
Iraq	39	37.1
Morocco	20	19
Algeria	17	16.2
Egypt	6	5.7
Palestine	5	4.8
Tunisia	5	4.8
Jordan	4	3.8
Syria	3	2.9
Lebanon	2	1.9
Kuwait	2	1.9
UAE	1	1
Oman	1	1
Years of practice		
1–10	57	54.3
11–20	22	21.0
21 and above	26	24.7
Mean ± SD	14.2 ± 10.9	
Range	1–41	
Total	105	100

### 3.2 Knowledge of biosimilars

Knowledge-related responses are summarized in Table 2. Respondents rated their overall knowledge of biosimilars at a mean of 3.74 on a 5-point Likert scale. Knowledge of the development process scored 3.33, and agreement that biosimilars reduce healthcare costs scored the highest at 4.36.

**Table 2:** Knowledge about biosimilars average weighted answers on a scale of (1–5).

Topic	Weighted average score
Knowledge of biosimilars:	3.74
Comfort with EMA/FDA approved biosimilar:	4.22
Comfort with non-EMA/FDA approved biosimilar:	2.65
Prescribe a biosimilar on treatment initiation:	3.73
Switch from an originator to a biosimilar when stable:	3.09
Switch to a biosimilar after poor response to originator:	3.14
Agreement that biosimilars reduce healthcare costs:	4.36
Knowledge of the biosimilars development process:	3.33
Concern about loss of clinical efficacy when switching to biosimilar:	3.65
Concern about adverse events when switching to biosimilar:	3.49
Concern about increased risk of immune reactions when switching to biosimilar:	3.57
Concern about loss of efficacy when switching from biosimilar to reference product:	3.35
Concern about adverse events when switching from biosimilar to reference product:	3.18
Concern about increased risk of immune reactions when switching from biosimilar to reference product:	3.25
Concern about loss of clinical efficacy among several biosimilars:	3.7
Concern about adverse events among several biosimilars:	3.58
Concern about increased risk of immune reactions among several biosimilars:	3.79

**Figure 1:**
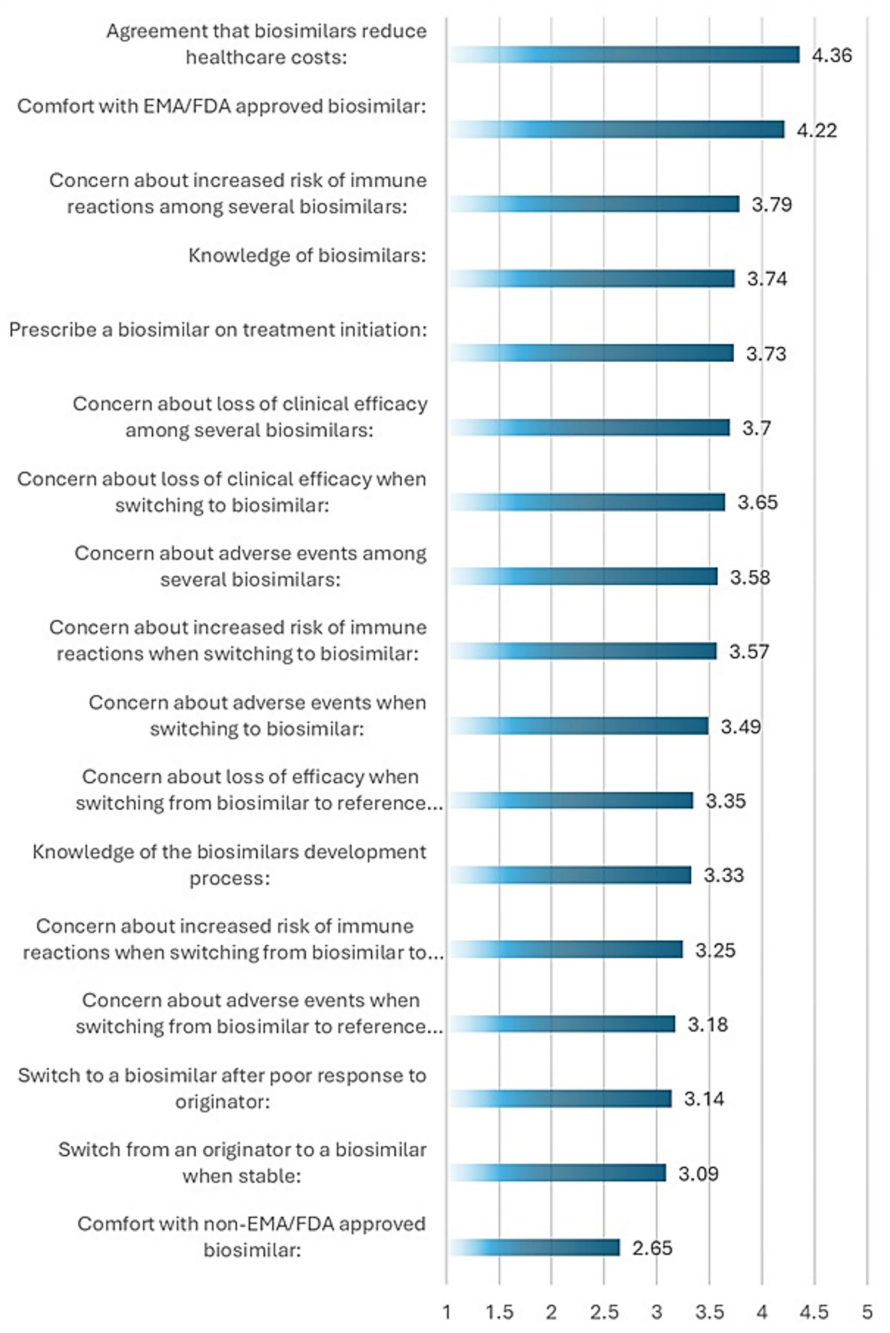
Mean Likert-scale scores (±standard deviation) for key survey items assessing rheumatologists’ knowledge, attitudes, and perceptions regarding biosimilars.

As illustrated in Figure 1, items related to belief in the cost-reducing potential of biosimilars showed high mean scores with low standard deviations, indicating broad consensus among respondents. In contrast, comfort with non-EMA/FDA-approved biosimilars and switching stable patients demonstrated greater variability, reflecting divergent perceptions across clinicians.

Objective assessment of knowledge is detailed in Table 3. When asked to select the most accurate definition of a biosimilar, 53 rheumatologists (50.5%) correctly selected the EMA/FDA definition: “a biological medicine that is highly similar to an approved biological medicine, with no clinically meaningful differences in safety and efficacy.” Following educational competency standards described by De Ketele and Gérard, knowledge was considered “good” when ≥75% of key responses were answered correctly (12). Additional detailed results regarding respondents’ understanding of specific evidence domains (Q11), perceived importance of evidence types (Q12), and EMA regulatory definition knowledge (Q17) are provided in Supplementary Tables S1–S3.

**Table 3:** Additional questions about biosimilars knowledge and use.

Question	Frequency	Percent
Which of the following most accurately describes a biosimilar?		
A biological medicine that is highly similar to an approved biological medicine, with no clinically meaningful differences in safety and efficacy profile	53	50.5
A biological medicine that is identical to an approved biological medicine, with identical safety and efficacy	27	25.7
A biological medicine that is similar to an approved biological medicine, but with more uncertain safety and efficacy profile	21	20.0
A biological medicine that is similar to an approved biological medicine, but with an improved safety and efficacy profile	4	3.8
Do you routinely use a biosimilar in your clinical practice to treat patients (excluding in clinical trials)?		
yes	90	85.7
No, because I’m not convinced/ comfortable about the use of biosimilars	9	8.6
No, for another cause	6	5.7
Where have you received information about biosimilar to date?		
Conferences/live meetings	63	60.0
Online education and/or self-study	42	40.0
Published literature	39	37.1
Colleagues	35	33.3
Other	6	5.7

### 3.3 Attitudes toward biosimilars

Attitudes toward prescribing biosimilars varied by clinical context (Table 2). Respondents reported high comfort with EMA/FDA-approved biosimilars (mean score: 4.22), but expressed less comfort with non-EMA/FDA-approved biosimilars (2.65). Willingness to prescribe biosimilars at initiation (3.73) was greater than willingness to switch after a poor response to the originator (3.14) or in stable patients (3.09). These trends highlight a generally cautious attitude toward switching, especially in patients with stable disease.

### 3.4 Perceptions of safety and immunogenicity

Concerns regarding safety and efficacy when switching to biosimilars are detailed in Table 2. Physicians expressed moderate concern about loss of efficacy (3.65), adverse events (3.49), and immune reactions (3.57). Concerns were similarly high when switching between multiple biosimilars or back to the originator.

### 3.5 Educational needs and priorities

As shown in Table 4, the most requested educational topic was comprehensive information covering all biosimilar aspects (39.0%). Among specific topics, FDA/EMA approval pathways were of greatest interest (24.8%), followed by product-specific comparisons (17.1%) and interchangeability/substitution (15.2%). On the topic of clinical trial design, 44.8% of respondents preferred endpoints that are most sensitive to detecting differences, rather than overall survival metrics. Regarding extrapolation of indications, 56.2% selected the correct regulatory definition, while 43.8% misunderstood extrapolation as use without supporting trial data. Encouragingly, 84 respondents (80%) expressed a desire for more ArLAR-led educational initiatives on biosimilars.

**Table 4:** Additional questions about biosimilars education.

Question	Frequency	Percent
What educational content related to biosimilars is of greatest interest to you?		
FDA/ EMA guidance and procedures for approval	26	24.8
Product-specific comparisons of biosimilars and reference products/innovators	18	17.1
Substitutions and interchangeability	16	15.2
Pharmacoeconomics	4	3.8
Comprehensive information (“All of the above”)	41	39.0
Which of the following endpoints do you think is most appropriate to use for studies of the comparative clinical efficacy of a biosimilar with a reference biologic?		
The endpoint considered most sensitive for detecting differences between the biosimilar and reference biologic, and least influenced by patient- or disease-related factors	47	44.8
The endpoint most strongly reflective of the clinical benefit of the biologic (e.g., overall survival or progression-free survival rates)	33	31.4
The primary endpoint that was used in the phase III trial with the reference biologic	25	23.8
Which of the following accurately describes the concept of ‘extrapolation of indications’ for biosimilars?		
Authorization of a biosimilar for use in an indication that is similar to one in which it has already demonstrated clinical comparability	59	56.2
Authorization of a biosimilar in indications of the reference biologic in the absence of specific clinical trial/data for the biosimilar in those indications	46	43.8
Would you like ArLAR to provide more educational activities in the biosimilars area?		
yes	84	80.0
No	13	12.4
I’m not sure if more education is needed on the topic	8	7.6

## 4 Discussion

**Figure 2:**
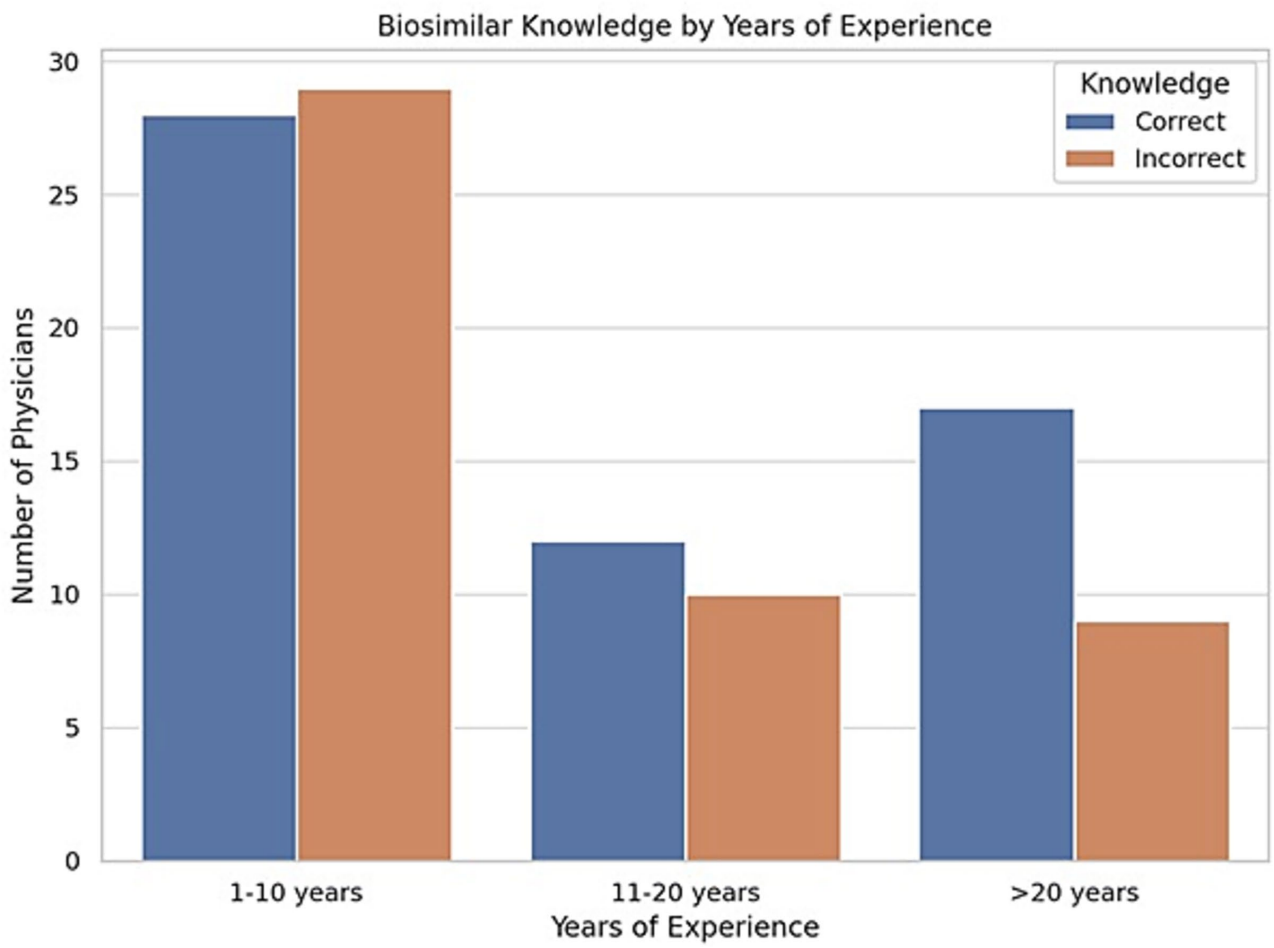
The distribution of biosimilar knowledge by years of clinical experience among rheumatologists.

**Figure 3:**
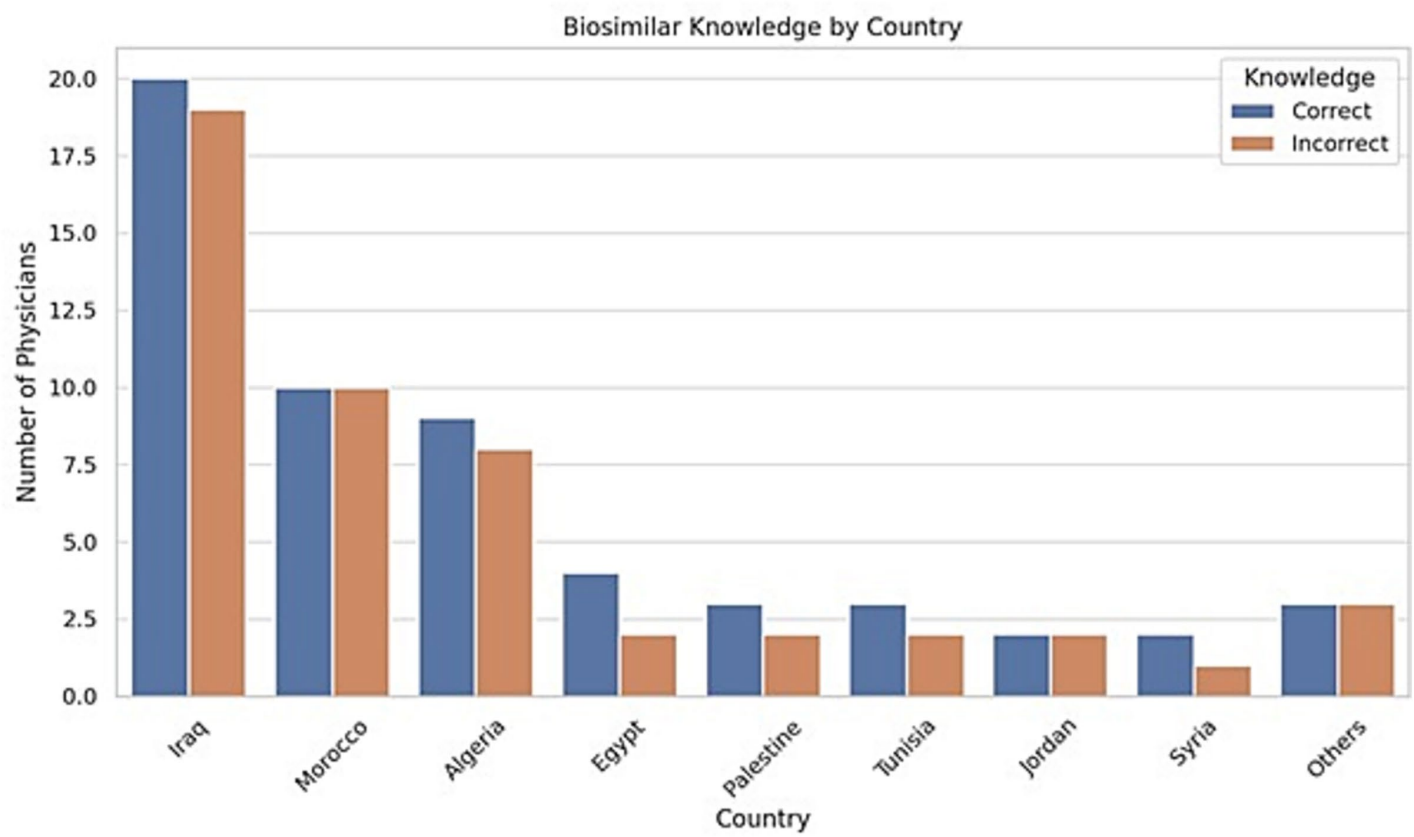
Biosimilar knowledge among rheumatologists by country.

This study presents one of the first regional assessments of biosimilar knowledge, clinical experience, and educational needs among rheumatologists in the ArLAR countries. The results show that while there is a generally favorable attitude toward biosimilar use, gaps in specific knowledge areas and concerns regarding safety, immunogenicity, and switching practices persist. Although the exact number of invitees is unknown due to the online dissemination method (email, WhatsApp, social media), we estimate the survey reached approximately 300–400 rheumatologists across ArLAR countries. This suggests a response rate of 25–35%. The finding that over 85% of respondents currently prescribe biosimilars in clinical practice is encouraging and aligns with global trends indicating increasing biosimilar adoption among rheumatologists (5, 6). However, this figure should be interpreted with contextual nuance. In certain ArLAR countries, physicians may have limited access to originator biologics during specific periods, and biosimilars may be used by necessity rather than as a clinical preference. This reflects the importance of considering both regulatory and procurement dynamics when evaluating prescribing behavior. The regulatory landscape for biosimilars across ArLAR countries is heterogeneous and reflects broader economic and healthcare system differences. Gulf Cooperation Council (GCC) countries generally operate under structured regulatory frameworks aligned with international agencies such as the EMA or FDA, supported by centralized procurement systems and expanding biosimilar pipelines (11). In contrast, several Levant and North African countries rely on mixed procurement models in which national tender systems and local manufacturing influence product availability (13). North African markets have experienced increasing penetration of locally manufactured or regionally approved biosimilars, while access in resource-limited or conflict-affected settings may remain variable. To provide additional context regarding regulatory oversight and product availability across the region, examples of commonly available biosimilars and their corresponding national regulatory authorities in selected ArLAR countries are summarized in Supplementary Table S4. These structural differences are consistent with previously reported variability in physician perceptions and biosimilar uptake across Arab countries (13). Physicians with >20 years of experience show the highest proportion of correct knowledge responses. Those with 1–10 years represent the largest group but with a lower proportion of correct responses (Figure 2). This trend suggests that clinical exposure may enhance biosimilar understanding, supporting targeted education for early-career rheumatologists. Iraq, Morocco, and Algeria had the highest participation and also the largest number of correct responses. Egypt, Palestine, and Tunisia had moderate representation with a good proportion of accurate knowledge. Countries grouped under “Others” showed varied but generally lower awareness, aligning with their smaller sample size (Figure 3).

This suggests that educational outreach is relatively more advanced in some North African and Levantine countries but still uneven across the ArLAR region, supporting the need for geographically tailored training efforts. Despite the high utilization rate, only 50.5% of respondents correctly identified the EMA/FDA-endorsed definition of a biosimilar. This suggests persistent misconceptions, with 25.7% of participants incorrectly believing biosimilars are “identical” to originator biologics. Such confusion has also been documented in international surveys and systematic reviews, which report variability in clinician and patient knowledge across regions (5, 14). This situates ArLAR-region knowledge levels within a comparable global context, highlighting that even in high-income countries, biosimilar literacy among clinicians remains suboptimal. These findings reaffirm that physician knowledge and confidence are central to the effective and ethical integration of biosimilars into routine care. Moreover, research suggests that a clinician’s ability to clearly explain biosimilar concepts can mitigate the nocebo effect, improving patient trust and adherence during switching (9). Analysis of Likert-scale responses provided additional insight into variability in physician perceptions (Figure 1). For instance, belief in the cost-reducing potential of biosimilars showed a high mean score of 4.36 ± 0.67, indicating strong agreement and minimal variability across respondents. Similarly, comfort with EMA/FDA-approved biosimilars was relatively consistent (4.22 ± 0.76), reflecting broad confidence in internationally regulated agents. In contrast, perceptions around switching stable patients to biosimilars (3.09 ± 0.91) and comfort with non-EMA/FDA biosimilars (2.65 ± 0.94) were more variable, suggesting that these topics remain areas of clinical uncertainty or differing institutional experience. Standard deviations exceeding 0.85 in these domains highlight greater heterogeneity in rheumatologist attitudes, which may reflect differences in national regulatory exposure, clinical experience, or access to specific biosimilar products. These patterns underscore the importance of targeted educational efforts that address nuanced aspects of biosimilar use—especially switching protocols and regulatory equivalence—to build confidence and reduce inter-clinician variability.

Addressing these gaps is particularly relevant for ArLAR countries, which are characterized by diverse healthcare systems, variable enforcement of regulations, and heterogeneous educational exposure (15). This underscores the value of regionally tailored, CME-accredited educational initiatives—particularly those emphasizing regulatory definitions, extrapolation principles, pharmacovigilance, and communication strategies (4). This mirrors findings in studies by Peyrin-Biroulet et al. and members of the EULAR taskforce, which showed greater acceptance of biosimilars when robust regulatory endorsement exists (14). Concerns about switching from originator to biosimilars, especially in clinically stable patients, remain moderate (mean score 3.09), and higher concern scores were noted for potential loss of efficacy and immunogenicity when switching among multiple biosimilars. This hesitancy is consistent with findings in the NOR-SWITCH and BIO-SWITCH studies, which demonstrated physician caution even when scientific evidence supported non-inferiority of biosimilars (16, 17). The desire for more educational content was strong, with 80% of participants requesting further initiatives by ArLAR. Importantly, the most requested topics were comprehensive overviews, regulatory frameworks (FDA/EMA approval), and clinical interchangeability—highlighting areas where future educational programming should be prioritized. Educational delivery preferences also varied, with conferences and live meetings being the most cited sources, reinforcing the importance of blended learning models that combine in-person sessions with online and literature-based approaches.

The knowledge gaps identified in this survey—particularly regarding the regulatory definition of biosimilars, extrapolation of indications, and switching in stable patients—underscore the need for structured, regionally adapted educational initiatives. Evidence from Europe suggests that society-led programs integrating regulatory science, interpretation of equivalence trials, and real-world switching data can improve clinician confidence and prescribing consistency (5, 18, 19). Given the comparatively lower knowledge levels observed among early-career rheumatologists in our cohort, incorporating biosimilar-focused modules into ArLAR congresses, fellowship curricula, and accredited CME activities may be especially beneficial. Ethical collaboration between pharmaceutical companies and professional societies may further strengthen biosimilar education if guided by transparency and scientific independence. Professional societies should lead content development and consensus guidance, while industry may support dissemination of up-to-date clinical and pharmacovigilance data under clear disclosure frameworks (20). From a policy perspective, higher-income ArLAR countries may benefit from formal prescribing guidance and pharmacoeconomic integration, whereas in lower-resource settings, strengthening regulatory harmonization and clinician education may have greater impact on adoption (21).

Several limitations of this study should be acknowledged. First, although the participant pool included rheumatologists from 13 ArLAR countries, the overall response rate was modest relative to the number of targeted clinicians, which may introduce non-response bias. Second, this was a self-reported, cross-sectional survey, and as such, the results may be influenced by social desirability bias and recall bias, particularly in questions related to knowledge or routine practice. Third, the online distribution format may have introduced selection bias, potentially favoring participation from younger rheumatologists or those more engaged with digital platforms and continuing education, who may also be more open to biosimilar adoption and innovation in general. Fourth, the relatively low representation from Gulf Cooperation Council (GCC) countries—which typically have higher healthcare expenditures, greater access to biologics, and more structured national regulatory systems—may have skewed results. This underrepresentation could limit the generalizability of findings related to biosimilar availability, procurement models, and physician autonomy in treatment decisions, particularly in wealthier Arab nations. Future studies should aim to improve geographic and institutional representation, potentially using mixed-methods designs (e.g., interviews or focus groups) to validate and expand upon survey findings. Fifth, subgroup differences by years of experience and country were not formally tested for statistical significance, as the analysis was primarily descriptive. Therefore, these findings should be interpreted cautiously, and future studies incorporating inferential statistics are needed to confirm these observations.

## 5 Conclusion

Rheumatologists across ArLAR countries demonstrate a positive overall attitude toward biosimilar use, with high levels of reported clinical implementation. Nonetheless, knowledge gaps remain, particularly regarding biosimilar definitions, development, extrapolation, and switching. These findings underscore the need for continued professional education to address misconceptions and build confidence, particularly in areas related to interchangeability and regulatory pathways. Tailored, multilingual, and regionally accessible educational programs are essential to optimize biosimilar integration into rheumatology practice in the Arab region.

## Data Availability

The raw data supporting the conclusions of this article will be made available by the authors, without undue reservation.
