# Supplementary material for: Perceptions and clinical use of biosimilars among rheumatologists in ArLAR countries: a cross-sectional survey
Source: Front Med (Lausanne). 2026 Mar 23;13:1780691. doi: 10.3389/fmed.2026.1780691 (PMC13050824; doi:10.3389/fmed.2026.1780691)
Supplement: Supplementary file 1 [file Data_sheet_1.docx]

**Perceptions and Clinical Use of Biosimilars Among Rheumatologists in ArLAR Countries**

**We would very much appreciate if you took 10 minutes of your time to complete the following survey.**

**I accept to participate □**

BACKGROUND/ Demographic data

**Age: __**

**Gender: □Man □ woman □ Prefer not to say**

**Years of practice in rheumatology specialty:_______**

**Country of practice: _______ ( Saudi Arabia, Bahrain, Iraq, United Arab Emirates, Egypt, Qatar, Jordan, Kuwait, Oman, Lebanon, Syria, Algeria, Morocco, Yemen, Sudan, Tunisia, Libya, Palestine, other)**

**Q1. Overall, how would you rate your knowledge of biosimilars? (1 = very low; 5 = very high)?_______**

**Q2. Which of the following most accurately describes a biosimilar?**

** A biological medicine that is identical to an approved biological medicine, with identical safety and efficacy**

** A biological medicine that is highly similar to an approved biological medicine, with no clinically meaningful differences in safety and efficacy profile**

** A biological medicine that is similar to an approved biological medicine, but with an improved safety and efficacy profile**

** A biological medicine that is similar to an approved biological medicine, but with more uncertain safety and efficacy profile**

**Q3. Do you routinely use a biosimilar in your clinical practice to treat patients (excluding in clinical trials)?**

** Yes  No, because I am not convinced/comfortable about the use of biosimilars  No, because biosimilars are not approved/reimbursed yet in my country**

**Q4. In principle, how comfortable are you with the concept of using a biosimilar, approved by EMA or FDA, to treat a patient suitable for the reference biologic? (1 = not at all comfortable; 5 = very comfortable)__-**

**Q5. In principle, how comfortable are you with the concept of using a biosimilar, not approved**

**by EMA or FDA, to treat a patient suitable for the reference biologic? (1 = not at all**

**comfortable; 5 = very comfortable)**

**Q6. If both an originator medicine and a biosimilar were available to you for prescribing, please indicate how strongly you agree or disagree regarding what you would carry out the following: (1 = strongly disagree; 5 = strongly agree)**

**□ Prescribe a biosimilar to a patient on treatment initiation**

**□ Switch from an originator medicine to a biosimilar when a patient is clinically stable**

**□ Switch to a biosimilar when a patient has had a poor clinical response to the originator medicine.**

**Q7. To what extent do you agree with the following statement: biosimilar medicines prescription allows reducing healthcare costs?’’** **(1 = strongly disagree; 5 = strongly agree)______--**

**Q8. Where have you received information about biosimilar to date? [Select all that apply]**

**□ Conferences/live meetings**

**□ Published literature**

**□ Online education and/or self-study**

**□ Colleagues**

**□ Other**

**□ No education on biosimilars to date**

Biosimilars Development and Evidence

**Q9. How would you rate your knowledge of the biosimilars development process and threshold of clinical evidence required for approval? (1 = very low; 5 = very high) _________-**

**Q10. What educational content related to biosimilars is of greatest interest to you?**

**□ Just the basics**

**□ FDA/ EMA guidance and procedures for approval**

**□ Product-specific comparisons of biosimilars and reference products/innovators**

**□ Substitutions and interchangeability**

**□ Pharmacoeconomics**

**□ Comprehensive information (“All of the above”)**

**Q11. How would you rate your understanding of the following kinds of data that are commonly generated for biosimilars? (1 = very low; 5 = very high)**

** Physicochemical data (e.g. molecular structure analyses, glycosylation data, etc.)**

** In vitro data (e.g. cell-based functional assays, receptor binding)**

** In vivo data (e.g. biological activity in animal models)**

** Pharmacokinetic data (e.g. Cmax, AUC, etc.)**

** Pharmacodynamic data (e.g. effect on pharmacodynamic markers)**

** Clinical efficacy and safety data**

** Immunogenicity data**

**Q12. Please rate the following in terms of how important you feel they are in determining the suitability of a biosimilar for use? (1 = not important; 10 = very important)**

** Physicochemical data demonstrating structural similarity**

** In vitro and in vivo data demonstrating similarity in biological activity**

** Pharmacokinetic and pharmacodynamic data demonstrating similarity**

** Clinical study data demonstrating similar efficacy**

** Clinical study data demonstrating similar safety**

** Clinical study data demonstrating similar immunogenicity**

** Clinical study data demonstrating the ability to switch from the reference biologic to the biosimilar and vice versa to the without impairing safety or efficacy**

**Q13. Which of the following endpoints do you think is most appropriate to use for studies of the comparative clinical efficacy of a biosimilar with a reference biologic?**

** The primary endpoint that was used in the phase III trial with the reference biologic**

** The endpoint considered most sensitive for detecting differences between the biosimilar and reference biologic, and least influenced by patient- or disease-related factors**

** The endpoint most strongly reflective of the clinical benefit of the biologic (e.g. overall survival or progression-free survival rates)**

Extrapolation of Biosimilars

**Q14. Which of the following accurately describes the concept of ‘extrapolation of indications’ for biosimilars?**

** Authorization of a biosimilar in indications of the reference biologic in the absence of specific clinical trial/data for the biosimilar in those indications**

** Authorization of a biosimilar for use in an indication that is similar to one in which it has already demonstrated clinical comparability**

**Q15. How would you rate your understanding of the requirements that need to be met for extrapolation of indications to be granted for a biosimilar? (1 = very low; 5 = very high)_____-**

**Q16. Biosimilar X has shown similar properties ,clinical efficacy and safety in one of the reference biologic’s indications. Provided that the biosimilar X was approved by EMA & FDA for all indications of the reference biologic, how comfortable would you feel in using biosimilar X in other indications that the reference biologic is approved for? (1= not at all comfortable; 5 = very comfortable)___________-**

Interchangeability and switching

**Q17. Which of the following definitions is incorrect, based on European Medicines Agency definitions?**

** Interchangeability = the possibility of exchanging one medicine for another medicine that is expected to have the same clinical effect**

** Switching = when the prescriber decides to exchange one medicine for another medicine with the same therapeutic intent**

** Substitution = the practice of dispensing one medicine instead of another equivalent and interchangeable medicine at pharmacy level, after consulting the prescriber**

**Q18. Please indicate how strongly you agree or disagree with the following statements regarding switching a patient from a biosimilar to a reference product (1 = strongly disagree; 5 = strongly agree)**

** I do not anticipate that switching will have a significant effect on the treatment benefit the patient receives from the product**

** I do not anticipate that switching will lead to emergence of additional adverse effects**

** I do not anticipate that switching will lead to harmful immunogenicity**

**Q19. How concerned are you about each of the following when switching a patient’s treatment from a reference product to a biosimilar ? (1 = not at all concerned; 5 = very concerned)**

** Potential loss of clinical efficacy**

** Potential for adverse events**

** Potential for increased risk of immune reactions**

**Would you like ArLAR to provide more educational activities in the biosimilars area?**

** Yes  No□ I am not sure if more education is needed on the topic**

**If you replied Yes above, please specify the type of educational activities that you would like ArLAR to organize.**

**Please feel free to add comments about the topics presented in this survey or any other topics related to biosimilar medicines
___________----**
