# Supplementary material for: Perceptions and clinical use of biosimilars among rheumatologists in ArLAR countries: a cross-sectional survey
Source: Front Med (Lausanne). 2026 Mar 23;13:1780691. doi: 10.3389/fmed.2026.1780691 (PMC13050824; doi:10.3389/fmed.2026.1780691)
Supplement: Supplementary file 2 [file Table_1.docx]

**Supplementary Table S1**

**Self-Rated Understanding of Biosimilar Evidence Domains**

(1 = very low; 5 = very high)

| **Evidence Domain** | **Mean ± SD** | **Median (IQR)** | **% High Understanding (≥4)** |
| --- | --- | --- | --- |
| Physicochemical data | 2.23 ± 1.14 | 2 (1–3) | 12.9% |
| In vitro functional assays | 2.15 ± 1.01 | 2 (1–3) | 8.1% |
| In vivo animal data | 2.23 ± 1.11 | 2 (1–3) | 12.9% |
| Pharmacokinetic data | 2.39 ± 1.08 | 2.5 (1–3) | 14.5% |
| Pharmacodynamic data | 2.58 ± 1.06 | 3 (2–3) | 16.1% |
| Clinical efficacy & safety data | 3.37 ± 1.06 | 4 (3–4) | 51.6% |
| Immunogenicity data | 3.05 ± 1.11 | 3 (2–4) | 32.3% |
