# Supplementary material for: Perceptions and clinical use of biosimilars among rheumatologists in ArLAR countries: a cross-sectional survey
Source: Front Med (Lausanne). 2026 Mar 23;13:1780691. doi: 10.3389/fmed.2026.1780691 (PMC13050824; doi:10.3389/fmed.2026.1780691)
Supplement: Supplementary file 3 [file Table_2.docx]

**Supplementary Table S2**

Perceived Importance of Evidence Types

(1 = not important; 5 = very important)

| **Evidence Type** | **Mean ± SD** | **Median (IQR)** | **% Rated ≥4** |
| --- | --- | --- | --- |
| Structural similarity | 3.22 ± 1.08 | 3 (2–4) | 36.5% |
| In vitro/in vivo biological similarity | 3.38 ± 1.11 | 3 (3–4) | 41.3% |
| PK/PD similarity | 3.40 ± 1.16 | 3 (3–4) | 46.0% |
| Clinical efficacy data | 3.83 ± 1.14 | 4 (3–5) | 52.4% |
| Clinical safety data | 3.83 ± 1.16 | 4 (3–5) | 55.6% |
| Immunogenicity data | 3.70 ± 1.12 | 4 (3–5) | 52.4% |
| Switching data | 3.71 ± 1.20 | 4 (3–5) | 52.4% |
