# Supplementary material for: Perceptions and clinical use of biosimilars among rheumatologists in ArLAR countries: a cross-sectional survey
Source: Front Med (Lausanne). 2026 Mar 23;13:1780691. doi: 10.3389/fmed.2026.1780691 (PMC13050824; doi:10.3389/fmed.2026.1780691)
Supplement: Supplementary file 4 [file Table_3.docx]

**Supplementary Table S3**

Knowledge of EMA Regulatory Definitions

| **Item** | **n (%)** |
| --- | --- |
| Correct identification of incorrect EMA definition | **45 (42.9%)** |
| Incorrect identification | **60 (57.1%)** |
| Total | 105 |
